# The Determinants of Panic Buying during COVID-19

**DOI:** 10.3390/ijerph18063247

**Published:** 2021-03-21

**Authors:** Grace Chua, Kum Fai Yuen, Xueqin Wang, Yiik Diew Wong

**Affiliations:** 1 School of Civil and Environmental Engineering, Nanyang Technological University, Singapore 639798, Singapore; chua0873@e.ntu.edu.sg (G.C.); cydwong@ntu.edu.sg (Y.D.W.); 2 Department of International Logistics, Chung-Ang University, Seoul 06974, Korea; xueqinwang@cau.ac.kr

**Keywords:** panic buying, health belief model, perceived scarcity, anticipated regret, COVID-19, health crisis

## Abstract

The COVID-19 pandemic has seen an unmatched level of panic buying globally, a type of herd behavior whereby consumers buy an uncommonly huge amount of products because of a perception of scarcity. Drawing on the health belief model, perceived scarcity, and anticipated regret theories, this paper formulated a theoretical model that linked the determinants of panic buying and analyzed their interrelationships. Subsequently, data were collated from 508 consumers through an online survey questionnaire in Singapore that was conducted during the early stage of the pandemic, before the onset of the circuit breaker in April 2020. Next, an analysis of the results was done through structural equation modeling. It showed that the effect of the health belief model dimensions (i.e., perceived susceptibility, perceived severity, outcome expectation, cues to action, and self-efficacy) on panic buying is partially mediated by the consumers’ perceived scarcity of products. Furthermore, the effect of perceived scarcity on panic buying is partially mediated by consumers’ anticipation of regret. This paper expands on the current theoretical understanding of panic buying behavior, giving insights into the possible measures and solutions that policymakers and relevant stakeholders can uptake to manage panic buying in future a pandemic or health crisis.

## 1. Introduction

COVID-19 has become a pandemic with its rapid spread around the world. The complexity in managing the public health crisis and easing the public’s fears is evident in the uncoordinated international response towards organizational and system-wide challenges [1]. An outcome of this highly uncertain situation is the unprecedented levels of panic buying worldwide [2,3]. This is exacerbated by “disaster capitalists” that exploit human-influenced and natural calamities through price gouging and profiteering via raised prices during supply or demand shocks [4]. New levels of toilet papers are being purchased in Hong Kong and Australia; many people are purchasing guns in the US; buying of groceries are at sky-high levels around the globe [5]. There has also been an increase in online and offline purchases [6,7]. This suggests that consumers will exploit all channels possible to panic buy.

Panic buying caused by the pandemic has severe negative effects on society. It creates negative externalities to society when perishable goods and household essentials are bought in excessive amounts and left to waste, depriving another consumer of consuming the goods [8]. Additionally, those panic bought items left to waste contribute to wasted energy and resource inputs utilized to produce them, leading to excessive greenhouse gas emissions [9]. Thus, this reduces the allocative efficiency of resources, leading to societal deadweight loss and potential stockouts. This detriment to society is even worse in lower-income countries with no social safety nets [10], as the negative effects of panic buying disproportionately fall on the disadvantaged. Psychologically, panic buying is more impactful on sufferers of mental health problems, substance-abusers or those in recovery, and people with reduced communication abilities [11]. Demographically, panic buying is also more detrimental to the elderly, the young undergoing education at a critical time, women caring for their family, those vulnerable to discrimination, homeless and people in the criminal justice system unable to self-isolate, undocumented migrants, workers on precarious contracts, and low-income people [11]. Overall, the panic buying situation has most severely hit goods that have been perceived as most essential and caused the greatest detriment on the most vulnerable segments of society.

As the COVID-19 pandemic is relatively nascent, the panic buying situation is a recurring phenomenon that has recently re-captured the public’s interest. Thus, the number of studies on this topic is very limited. However, business activities and consumer buying patterns have undeniably been impacted by COVID-19 [12,13]. Research on panic buying can be categorized based on the cause and effect of panic buying.

Determinants of panic buying include social proof and influence from close relations. Social proof is an increasingly crucial source of psychological and social signal that influences people to select the option chosen by the majority [14], triggered by collaboratively shared information and experiences of others [15,16,17,18]. The actions of close relations can influence an individual to engage in panic buying [19]. Social influence from these close relations can impact an individual’s attitude, subsequent behaviors, and actions, affecting their decision to panic buy. Additionally, social media sharing of pictures and videos of panic buying behaviors and outcomes have motivated panic buying. Furthermore, individuals’ panic buying behavior is also influenced by the contagion model, perception of severity, social-psychological factors, fear of the unknown, coping instinctual behavior, social learning theory, trust in government and media, media content, agent-based model, and the stimulus-organism-response framework [20,21,22,23,24,25]. Finally, risk-averse individuals show a tendency to copy the behaviors and attitudes of people that they perceive to be similar to themselves [19]. This is especially relevant to Singapore, identified to be one of the most uncertainty avoidant country in the world [26,27], because Singaporeans are less tolerant to risks and ambiguous situations.

The research on the effects of panic buying is slightly more robust. They include the effects on supply chain disruptions and purchasing behavior of retailers, subsequent cascading effect of panic buying, effects on pharmacy purchases of drugs from wholesalers, effects on online grocery shopping [28], effects on consumer panic levels [29], effects on transport volume and freight capacity dynamics, and effects on the primary, secondary, and tertiary industries [7,30,31,32]. These effects range from supply chain disruptions and concerningly low levels of animal pharmaceuticals needed by primary agricultural industries to volatile changes in freight volume for retail logistics dependent on the strength of the pandemic, as measured through the number of new daily infections.

At present, no study has analyzed the causes of panic buying from a belief viewpoint. A belief is the inner convictions, concepts, values, and precepts that an individual believes to be correct [33]. The effectiveness of beliefs in justifying behaviors is commonly accepted in psychology research [34,35]. An appropriate belief model that can be used to describe panic buying is the health belief model. This model was initially applied to draw links between health behavior and undergoing routine medical examinations. It highlights those personal perceptions (i.e., perceived severity and susceptibility), modifying factors (i.e., self-efficacy and outcome expectations), and actions (i.e., cues to action) that justify a behavior [36]. In the context of panic buying, it is relevant because it studies the protection motivation behavior of consumers when faced with the threat of a disease outbreak. As the COVID-19 crisis disrupts supply chains globally, stockout and shortage situations seem to be increasing in scale and frequency especially for certain drugs [37]. Protection motivation behavior is the activity engaged in to protect oneself from a threatening event based on one’s perception of four considerations: severity of the dangerous situation, likelihood of the incidence of danger, benefits of the suggested preventive behavior, and personal ability to adopt the behavior. It is a threat and coping appraisal pathway used to explain the intention to engage in a protective preventive behavior [38]. Thus, panic buying is a consumer behavior seen as effective in preventing oneself from experiencing a stockout of essential products by ensuring an available inventory of personal supply. Additionally, panic buying large amounts of supplies is also a method of protecting consumers from contracting COVID-19 because it lowers the risk of infection by reducing the frequency of shopping trips and exposure to external environments, thus reducing their susceptibility to contracting COVID-19.

However, this study argues that the health belief model does not fully account for panic buying behavior. Thus, this study incorporates perceived scarcity and anticipated regret theories to understand panic buying from a more comprehensive causation perspective. Currently, very few studies on panic buying have analyzed the phenomenon from the perceived scarcity and anticipated regret perspectives. A perception of scarcity is highly correlated to panic buying especially if the scarcity develops for the immediate necessities [39,40,41]. Furthermore, in the realm of rational choice behavior, the anticipated regret is a key factor when trying to achieve an optimal decision. In the context of panic buying, consumers may anticipate that if they do not buy their necessary items now, they may regret it later. Thus, consumers may believe that the optimal decision to minimize regret later would be to purchase more at the present moment.

To address the research gap, this study’s objective is to draw on the theoretical contributions of the health belief model, perceived scarcity, and anticipated regret theories to understand the elements affecting the panic buying behavior and study their interrelationships. This paper posits that the health belief model factors (perceived susceptibility, perceived severity, outcome expectation, and cues to action) influence the perceived scarcity of products, which in turn affect consumers’ anticipated regret and panic buying behavior. Further, panic buying behavior is also directly influenced by consumers’ anticipated regret. Having studied the determinants of panic buying, the paper strives to analyze how government intervention can overcome free-market capitalism failure in the context of COVID-19.

Through structural equation modeling, the theoretical model as composed of eight constructs is estimated. This method is chosen to increase the theoretical model’s estimation accuracy given the multi-dimensional and unobservable latent constructs present.

The remaining sections of this paper are written in the following manner. The first section summarizes the theories used to synthesize the model applied to explain the panic buying behavior. Here, relevant conference papers, journal articles, and newspaper articles are reviewed. Subsequently, an overview of the data collection methodology is given. This is followed by an analysis of the survey results collected. Lastly, based on the results, policy implications and the research agenda for further study are offered.

## 2. Literature Review

### 2.1. Theory and Model

This research employs three concepts to describe the determinants of panic buying. They are the health belief model, perceived scarcity, and anticipated regret theories. Based on psychology, these constructs analyze the cognitive, affective, and behavioral aspects of panic buying. Table 1 summarizes the theories’ fundamental assumptions, underlying concepts and relevancy to the model design.

**Figure 1:**
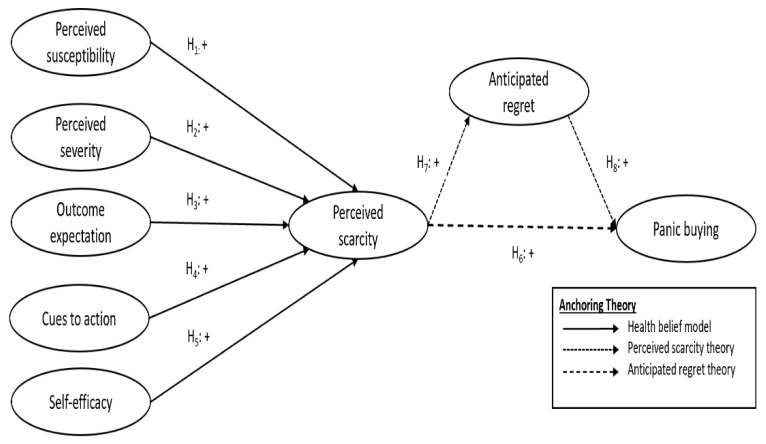
The proposed structural equation model.

As shown in Figure 1, the theoretical model presents the interrelationships of the constructs and panic buying behavior. The health belief model has been applied in the numerous community-based health interventions contexts [42,43,44] and its elements (i.e., perceived susceptibility, perceived severity, outcome expectancy, cues to action, and self-efficacy) explain the determinants of the perceived scarcity of products in the pandemic. This perceived scarcity subsequently leads to anticipated regret and panic buying behavior [52]. The health belief model is traditionally used to identify the reasons for people’s failure to implement disease prevention procedures or screening examinations for early disease detection [55] and has been shown to influence the adoption of health prevention behaviors [56]. It is a widely accepted model in the health behavior field to justify the change and maintenance of health-related interventions [43,48,49,57,58]. In the context of the current study, panic buying can be considered as preventive health behavior, which helps to contain the chance of being infected by COVID-19 [59] and affected by a stockout of goods and services due to supply chain disruptions.

The health belief model’s factors are posited to impact consumers’ perceived scarcity of goods and services because they influence consumers’ understanding of the availability of resources when making shopping choices. Essentially, perceived susceptibility and severity (i.e., perceived vulnerability to and risk of contracting COVID-19), as well as self-efficacy in protecting oneself from the pandemic are directly related to consumers’ level of worry, task, and response orientation [60] towards ensuring the availability of goods. Thus, consumers perceive scarcity when they perceive the susceptibility (H_1_) and severity (H_2_) of contracting COVID-19 to be high. This perception of scarcity is also increased when the outcome expectation (i.e., expected functional utility and ease) of panic buying is high (H_3_) and cues to action (i.e., social influence, news reports, and social media) to panic buy (H_4_) are high too. Lastly, consumers’ perception of scarcity will increase when consumers have high self-efficacy in protecting themselves against COVID-19 and coping with the pandemic (H_5_).

Perceived scarcity can be defined as an individual’s conception of limited availability [61]. This induces consumers to increase the amount they purchase due to an increased urgency or perceived value of the good [62]. This could be due to a risk of losing one’s freedom and can prompt heightened awareness and interest towards the inaccessible product, increasing one’s incentive to acquire the close substitute that may be unavailable soon [63,64,65]. The reactance, a psychological motivational state triggered by the belief that their freedom when carrying out a certain behavior is restricted [66,67], may encourage panic buying behaviors (H_6_) [52,53]. This is because consumers may respond swiftly and sometimes irrationally, to the perceived scarcity, to rebuild forgone freedom [68].

This study posits that perceived scarcity has a direct impact on anticipated regret consumers experience (H_7_) [51]. Anticipated regret is the consideration of the negative emotion, regret, when deciding to carry out an action to prevent an unwanted outcome (i.e., a future stockout) [45,46,47]. There is a high level of anticipated regret, a foreseen psychological opportunity cost leading to negative feelings, for not making an immediate purchase of limited goods especially when there is a perception of scarcity. In individuals with higher anticipated regret, their higher conception of health risk leads them to partake in activities they believe to minimize their risks [69,70]. Thus, this might trigger panic buying. This intention to panic buy will be reinforced if consumers believe that panic buying reduces the anticipated regret of unwanted outcomes, such as not having enough during stockouts or contracting COVID-19 (H_8_).

### 2.2. The Determinants of Perceived Scarcity

In this study, perceived scarcity is the consumers’ conception of the degree of resource abundance or availability in this COVID-19 pandemic. There are limited papers that have analyzed the determinants of consumers’ perceived scarcity leading to panic buying.

Existing research suggests that perceived scarcity is influenced by a combination of a loss of control over the surrounding environment [41], the feeling of insecurity and instability [71], and supply chain interruptions [72]. As social creatures, consumers are affected by herd instinct (i.e., the level of intensity of reactions by those around us) in their perception of scarcity [39,40] and this is exacerbated by the narrative from the media [73], which may exaggerate the threat of contracting COVID-19. Additionally, perceived scarcity is influenced by the primitive part of our brains becoming more prominent to impact our judgements and decisions necessary for survival [74]. Furthermore, perceived scarcity is influenced by consumers seeking the benefits of reduced uncertainty and anxiety as well as an increased sense of control [75]. Finally, perceived scarcity is influenced by the vulnerability consumers perceive themselves having due to the lack of confidence in the government, leading to the overestimated probability of danger and underestimated possibility of help [41].

Therefore, the aforementioned determinants adhere to the perceived scarcity components, where perceived scarcity is influenced by the perceived vulnerability, perceived threat, perceived advantages, social cues, and survival-based decision making. These determinants holistically touch on all five of the health belief model components (i.e., perceived susceptibility, perceived severity, outcome expectation, cues to action, and self-efficacy). Furthermore, cues to action and self-efficacy are motivational constructs that are believed to enhance the model’s explanatory power [76,77,78,79,80]. Thus, the elements of the health belief model are put forth to provide a more holistic, concise, and effective justification for the development of consumers’ perception of scarcity during the disease outbreak. The subsequent segments examine the effect of these components on consumers’ perceived scarcity.

#### 2.2.1. The Impact of Perceived Susceptibility on Perceived Scarcity

This study terms perceived susceptibility as consumers’ perception of risk or the chance of contracting COVID-19. Their level of perceived vulnerability may be dependent on their physiological and psychological health state in conjunction with their confidence in the management of the pandemic in the future. In other words, it refers to the probability of contracting COVID-19 because of the consumers’ vitality or their faith in the state of the future given the pandemic situation. Perceived susceptibility is a strong motivating force behind health-related behavior [81].

Consumers will perceive scarcity to a greater extent if the perceived risk of contracting COVID-19 is high. Generally, if the risk of contracting COVID-19 is greater, then the chance of restrictions on freight movement will increase, slowing the rate of replenishment of supply chain inventory. Thus, reducing the availability of products, leading to more frequent stockouts, and creating the perception of scarcity.

**Hypothesis** **1 (H1). ** *Perceived susceptibility of contracting COVID-19 has a positive impact on consumers’ perception of scarcity.*

#### 2.2.2. The Impact of Perceived Severity on Perceived Scarcity

This study terms perceived severity as the degree of detriment suffered from contracting COVID-19. This measures the conception consumers have towards the impact of contracting COVID-19 towards one’s career, relationships with friends and family, financial aspect, and one’s future in life. It also affects the level of fear one experiences when faced with the prospect of contracting COVID-19.

The disease outbreak has led to psychological risk factors such as fear, depression, anxiety, and stress of getting infected by COVID-19 [82,83,84]. Educational stress, joblessness, relationship troubles, poverty are also common underlying psychiatric problems [82,83,84,85]. During crisis periods such as this current disease outbreak, consumers are likely to unthinkingly exaggerate the threat and underestimate the probability of receiving help [41]. The research by Dsouza et al. (2020) highlighted that the anxiety of contracting COVID-19 (*n* = 21) is the most significant suicide causation than the financial crisis (*n* = 19). Societal rejection, family disputes, and the burden of being unable to go back to one’s homeland were also significant risk considerations [86,87].

These negative psychological, economic, social, and emotional impacts from the pandemic may lead to consumers losing confidence in the future, causing them to expect the worst-case scenario situations. Thus, consumers’ weak faith in the future may lead them to believe that there will likely be a collapse of the supply chain and perceive an increase in socially undesirable behaviors in other consumers (i.e., panic buying, hoarding, and stockpiling) to protect themselves rather than look out for the interest of others. Overall, this eroded trust in the reliability of the supply chain, society, and other community members may lead to consumers believing that there will likely be more frequent stockout situations and increasingly limited availability of goods and services. Thus, the common factors underlying the perceived severity of contracting COVID-19 may increase the perceived scarcity of products [21].

**Hypothesis** **2 (H2).** *Perceived severity of contracting COVID-19 has a positive impact on consumers’ perception of scarcity.*

#### 2.2.3. The Impact of Outcome Expectation on Perceived Scarcity

This study terms the outcome expectation of panic buying as comprised of perceived benefits and perceived barriers. It is the perceived utility that consumers expect themselves to gain from the act of panic buying, net of the loss in utility they expect themselves to lose due to the perceived barriers of panic buying. Outcome expectation will be positive if perceived benefits offset the perceived barriers of panic buying. Following the same logic, outcome expectation will be negative if perceived barriers outweigh the perceived benefits of panic buying. The types of benefits include protection from a stockout situation, lower likelihood of contracting COVID-19, and the feeling of security and safety. The perceived utility can be classified into four main categories (i.e., economic, hedonic, functional, and social utility) [88].

In terms of economic utility, panic buying overcomes the problem of price uncertainty driven by increased perceived consumer competition during the COVID-19 pandemic [89]. Panic buying ensures that consumers buy goods at the current price instead of subjecting themselves to the volatility of price fluctuations in the future. Furthermore, those who panic buy have the option to re-sell products at a profit if demand greatly exceeds supply [11]. This is especially prevalent for essential goods.

In terms of hedonic utility, panic buying can provide consumers with a feeling of purpose and control especially during periods of high ambiguity. Panic buying allows consumers to focus their energy on gaining control over their lives and reduces their anxiety and stress levels [3,21]. Panic buying may appeal to specific demographic clusters that are relatively more susceptible to contracting COVID-19. The notion of frequent external contact with the virus may cause them great anxiety and fear, thus causing them to favor panic buying. Additionally, given that panic buying saves time for consumers, the saved time can be apportioned to receiving entertainment.

In terms of functional utility, panic buying is a safer option than risking stockouts in the future. Given the supply chain disruptions and erratic consumer behavior during the pandemic, the risk of stockouts has greatly increased [21,37,51]. From the perspective of increasing personal safety by limiting exposure to COVID-19, panic buying allows consumers to minimize external contact, thus increasing their level of safety. It is also more time-efficient and convenient as it reduces consumers’ commuting and shopping time. Thus, the savings in the efficiency of each shopping window increases the functional utility of panic buying.

Finally, in terms of social utility, panic buying is a form of obtaining social identity and acceptance. With social media, consumers are increasingly connected through the sharing of information online [90,91]. They are kept informed via online ratings, advertisements, online forums, and social network influencers [92,93]. With more content of their peers’ panic buying being shared on social media, consumers seek a sense of belonging and shared identity with their peers by joining in the panic buying trend. Additionally, given that many people are kept in their homes because of COVID-19, social acceptance and social identity gained through social media are increasingly important. Thus, panic buying leads to an increase in social utility through an increased sense of social identity.

Therefore, the positive economic, functional, and hedonic utility of panic buying often leads to consumers choosing to panic buy more than necessary to maximize the outcome expectation of panic buying. The perceived benefit component of outcome expectation is recognized as the most powerful predictor of behavior [55,77,94,95,96].

Perceived barriers are obstacles consumers think may prevent them from enacting the panic buying behavior. The possible perceived barriers include the view that panic buying is a socially undesirable action (i.e., negative social utility) and makes them a mockery to their peers. This perceived barrier effect is especially strong if overall perceived barriers (time, health, social, and money-perceived barriers) of panic buying exceed the overall perceived benefits of panic buying.

In general, an increase in an overall positive outcome expectation of panic buying can lead to the perception that the other consumers will also seek to panic buy more so as to reap these perceived benefits. Thus, it leads to the perception of scarcity amongst consumers.

**Hypothesis** **3 (H3).** *Outcome expectation of panic buying has a positive impact on consumers’ perception of scarcity.*

#### 2.2.4. The Impact of Cues to Action on Perceived Scarcity

This study terms cues to action such as historical events (past experiences), mass media, and social influence (family, friends, neighbors, and colleagues) as activators for consumers’ readiness to panic buy. Consequently, these cues will have an impact on consumers’ perception of the scarcity of goods [95,97,98].

Cues include external and internal stimuli. External stimuli such as broadcasting networks (online news, social networking services, and television) can give a considerable prediction of risk perceptions around food risks concerning consumer’s health [99]. Additionally, social learning theory indicates that consumers tend to follow the herd instinct [22]. Internal cues such as past trauma with the related commodity may lead to a stronger perception of scarcity for that commodity [100]. For example, past encounters with water scarcity and deep-seated distrust of water security can lead to a perception of water scarcity [101]. Thus, external social cues of increased panic buying of limited stock from media and community members as well as internal cues from past personal experiences of food insecurity may cause consumers to increase their conception of limited availability of goods and services. Thus, external and internal cues to action can increase consumers’ perception of scarcity.

**Hypothesis** **4 (H4).** *Cues to action to panic buy has a positive impact on consumers’ perception of scarcity.*

#### 2.2.5. The Impact of Self-Efficacy on Perceived Scarcity

This study terms self-efficacy as a consumer’s perceived ability to protect oneself from COVID-19 and cope during the pandemic. It dictates the sufficiency of a consumer’s self-management mechanisms and proclivity to protect oneself from failure events [102,103]. This is because people who experience lower self-efficacy undermine their capacity to manage a vast variety of problems and have weaker coping mechanisms to manage their social anxiety and stress [104].

Therefore, an increase in self-efficacy in protecting oneself from COVID-19 and coping with the pandemic would lead to consumer’s propensity to manage their stress and anxiety through proactively taking precautions to protect themselves from the pandemic. Consequently, this leads to escalated worrying about the limited availability of products remaining, leading to the perception of scarcity of goods. Thus, high levels of self-efficacy would raise the perception of scarcity.

**Hypothesis** **5 (H5).** *Self-efficacy in protecting oneself from COVID-19 has a positive impact on consumers’ perception of scarcity.*

### 2.3. The Direct Impact of Perceived Scarcity on Panic Buying

This study terms perceived scarcity as an individual’s conception of a product’s limited availability, leading to the expectation that the product will become inaccessible soon due to the COVID-19 pandemic crisis.

According to the reactance theory, a product’s perceived scarcity implies a threat of personal freedom which triggers psychological reactance which raises consumers’ incentive to acquire the substitute that may be unobtainable soon [63,64,65]. Perceived scarcity is posited to be influenced by the loss of freedom (i.e., prohibited or diminished access to product) [105,106]. The perceived loss of freedom increases the perceived attractiveness of the limited goods and services [107], leading to an increased yearning to realize the prohibited actions (i.e., panic buying the limited goods and services). Additionally, the greater the perception of scarcity, the more unprotected and vulnerable to a situation consumers feel they are to contracting and suffering the consequences of COVID-19, the more likely they are to participating in protective measures to avoid the danger [108]. Thus, they are more likely to panic buy to minimize the frequency of their shopping trips and the risk of contracting COVID-19. This psychological reactance leads to heightened consumer motivation to panic buy the products that are perceived to be scarce [109,110].

**Hypothesis** **6 (H6).** *A perceived scarcity of goods and services has a positive impact on panic buying*.

### 2.4. The Indirect Impact of Perceived Scarcity on Panic Buying

This study also proposes that perceived scarcity will have an indirect impact on panic buying via anticipated regret. Regret is an affective and cognitive element that comprises the following components: it is unpleasant and preferably avoided, it is unlike other undesirable feelings, and it entails counterfactual reasoning [111]. Anticipated regret is the consideration of the negative emotion, regret, being taken into account when making a choice in an attempt to avoid the unwanted outcome [45].

In this pandemic outbreak context, the perceived scarcity event is likely to be a conception of a demand-driven limited-quantity scarcity event. Thus, perceived scarcity will likely lead to an increase in perceived consumer rivalry and perceived price insecurity for available inventory. This raised perception of rivalry and price insecurity will likely increase anticipated regret [51] amongst consumers because consumers will foresee themselves regretting if they do not successfully outrun other consumers rushing to stock up on the goods in limited supply before they are stockout. From the price uncertainty perspective, they may foresee themselves regretting if they do not purchase goods at the current lower price before the price spikes [112].

Overall, the foreseen risk of not being able to purchase products in limited supply and missing out on products being sold at lower prices triggers this anticipated regret.

**Hypothesis** **7 (H7).** *Perceived scarcity has a positive impact on anticipated regret.*

During high uncertainty, consumers anticipate regret if they fail to panic buy while they still could [67]. This anticipated emotion evolves from a rejected option (i.e., panic buying). Regret is felt if the rejected choice (panic buying) results in being better than the actual outcome [21]. This may be because consumers regret paying a higher price during a later purchase relative to the price they would have paid if they purchased it earlier in the form of panic buying. Alternatively, consumers may regret experiencing a stockout situation because they did not panic buy earlier when given the choice.

In resonance with the prospect theory, which describes decision-making behaviors in uncertain circumstances such as loss prevention, consumers will more probably feel regret rather than cheer for not panic buying due to perceived scarcity [113]. In anticipation of the uncertain future, consumers would rather take preventive action and panic buy to prevent future regret of paying a higher price or experiencing a stockout scenario. In past studies, anticipated regret has been reported to enhance intentions [114,115] or strengthen the connection between present intentions and behaviors [116,117]. Furthermore, anticipated regret is a construct that has a moderate to strong correlation with the behavioral intention of panic buying [46,54]. Thus, anticipated regret for failing to panic buy reasonably enhances the intention to panic buy when faced with a risky perceived scarcity event, increasing the likelihood of the actual panic buying behavior.

**Hypothesis** **8 (H8).** *Anticipated regret for failing to panic buy has a positive impact on panic buying.*

## 3. Methodology

To analyze the data, structural equation modeling is used to assess the theoretical model. It is chosen because of three motivations. To begin with, it permits the assessment of a theoretical model comprising many dependent constructs from various theories (i.e., health belief model, perceived scarcity theory, and anticipated regret theory). Secondly, the latent constructs are multi-dimensional and unobservable and need to be measured by observable variables, so structural equation modeling caters to measurement error, increasing the model estimation’s accuracy. Most crucially, compared to regression analysis, correlations with the endogenous constructs can be estimated simultaneously using structural equation modeling.

This study applied the Checklist for Reporting Results of Internet E-Surveys (CHERRIES) framework to design and administer its survey. All information and procedures are reported in the Appendix A.

### 3.1. Measurement Items

As the analysis of latent constructs is required in this paper, measurement items need to be included to operationalize each construct (Table 2). To operationalize consumers’ perceived susceptibility towards contracting COVID-19, three key measurement items were surveyed, namely the consumer’s perceived chance of contracting COVID-19 relative to others, the chance of contracting COVID-19 due to physical health, and the chance of contracting COVID-19 in the future [118]. This was assessed based on a scale ranging from 1 (extremely low) to 7 (extremely high).

To assess consumers’ perceived severity of contracting COVID-19, three measures were modified from [119] to show both personal (i.e., the degree of influence contracting has on the individual (SEV1) and their relationships with family and friends (SEV3)) and professional dimensions (i.e., the degree of influence contracting COVID-19 will have on the individual’s career (SEV2)). To some extent, the measures reflect the definition of perceived severity which concerns the conception consumers have towards the impact of contracting COVID-19 towards one’s career (SEV2), relationships with friends and family (SEV3), financial aspect (SEV2), and one’s future in life (SEV2 and SEV3).

Another three measures were modified from [119] to operationalize consumers’ outcome expectation of panic buying. As seen in Table 2, the measures reflect consumers’ perception of the benefits they reap currently (i.e., minimal exposure to external crowds) and in the future (i.e., potential stockout situation). Accordingly, the consumers’ outcome expectation of panic buying is measured by the positive utility they place on panic buying in its totality.

Three measures were obtained from [120] who proposed internal personal factors (i.e., SEL1 and SEL2) and external environmental factors (i.e., SEL3) in assessing the level of self-efficacy (i.e., the confidence and knowledge to protect oneself from COVID-19).

To assess cues to action, four measures were modified from [119]. The measures selected represent different sources of social influence that trigger panic buying such as influence from family (CUE1), previous experience (CUE2), friends (CUE3), and media (CUE4). These measures have proven to be valid and reliable in the context of health-promoting behaviors [119] and are thus adopted in this study.

To measure the perceived scarcity of consumers, three measures were adapted from the research by [121]. From the perspective of the availability of different products, three measures have been analyzed based on the product type (SCA1), brand (SCA2), and size (SCA3).

Anticipated regret is the anticipated loss of not buying. The level of anticipated regret increases with perceived perishability [122]. It can be measured based on a consumer’s foreseen emotions (i.e., REG1 and REG2) and self-constructs (i.e., REG3).

Lastly, panic buying can be measured based on consumers’ tendency and compulsion to panic buy. This is reflected in the thought process (i.e., internal urge to pick up an item) and actions (i.e., tendency to pick up an item and willingness to put down an item) of consumers.

### 3.2. Survey Design and Administration

The survey consists of three sections. The first section introduced the survey respondents to the rationale and goal of the research. The questionnaire also guarantees the participants that their identities will not be disclosed under any circumstance and encourages honest responses to the questions posed. Subsequently, the participants were prompted to recall their first visit to the local grocery store after the start of the circuit breaker (i.e., lockdown) as a precursor to answering the survey questions. Section two collates the participants’ demographic profile (i.e., gender, age, education, housing, household income, and online shopping frequency). Lastly, section three consists of all the measurement items stated in Table 2. Identical items but in a reversed manner are included in the survey to validate the participants’ responses.

A professional survey firm was engaged to administer the online questionnaire on a panel of respondents. A blended partnering panel approach was taken to ensure that the sampling frame is representative. In the beginning, the survey underwent a soft launch, whereby a small sample of responses was collated, to make minor improvements to the survey. Afterward, the adjusted survey questionnaire was then officially administered and 508 valid responses were collected. In the end, the company was given a lump-sum payment, with a fraction of it being used as monetary incentives to reward the qualified respondents.

### 3.3. Respondents’ Demographics

The 508 questionnaire participants’ demographic profiles have been highlighted in Table 3. The ratio of male (51%) to female (49%) participants was representative as the sample percentage split is relatively comparable to Singapore’s population gender split of 51.1% male to 48.9% female [127].

Next, nearly half of the sample earned more than SGD8000/year (45%) and are above 35 years old (51%). About 78% of the sample’s housing type is public housing, which is similar to the population’s average value of 80% [128].

Furthermore, about 85% of the sample received tertiary education. This statistic reflecting a sizeable proportion of the sample to be highly educated corresponds to the country’s focus on education. In 2019, 55.8% of Singaporeans from 25 to 29 years old had at least a graduate degree [129].

Additionally, Rakuten Insights found that 3% of Singaporeans shop online on a daily basis, while 30% of the population shops online a few times a month [130]. This is similar to the participants’ online shopping frequency proportions collected in this study. Thus, the above comparisons ensure the validity of the sample’s representativeness.

## 4. Results and Discussion

Structural equation modeling comprises the measurement model analysis (i.e., confirmatory factor analysis) and structural model analysis [131]. The first analysis (measurement model analysis) is to study the interrelationship among the constructs and their measurement items (i.e., factor loadings) while the second analysis (structural model analysis) is to study the interrelationship among the constructs derived from the hypotheses. Furthermore, to identify omitted, significant structural paths, a post hoc analysis is conducted. Lastly, the analysis on the direct, indirect, and total effects are used to understand the wider impact on theory-based and policy research.

### 4.1. Measurement Model Analysis

The measurement model analysis results are shown in Table 4. The fit indices satisfy the minimum benchmark suggested by Hu and Bentler (1999). To illustrate, the Tucker-Lewis index (TLI) and comparative fit index (CFI) exceed the suggested 0.95 while standardized root mean square residual (SRMR) and root mean square error of approximation (RMSEA) fall below 0.10 and 0.08, respectively. Thus, meeting the satisfactory model fit needed by the measurement model.

Table 4 shows the high reliability of the measurement items. Additionally, the measurement items’ factor loadings and composite reliabilities are mostly more than the advised value of 0.70 and 0.80 [131]. Table 5 affirms the validity of the measurement items. Firstly, there is convergent validity because each construct has an average variance extracted (AVE) of more than the advised 0.50 value [131]. Additionally, the discriminant validity, established as the AVE from each construct pairings, is more than their squared correlations. Cross sectional survey data tend to present common method bias. Thus, the Harman single factor test is used to analyze the common method bias. If the total variance extracted by a single factor is below 0.50, there is minimal common method bias. Therefore, at 0.37, there is little common method bias.

### 4.2. Structural Model Analysis

To analyze the other constructs’ correlations that were overlooked, a post hoc analysis, a data-driven method examining the modification index (MI), was done. Based on the principle of parsimony, two statistically significant paths (i.e., χ^2^ > 3.841, *df* = 1) were sequentially added. The sequence of adding the paths starts with introducing the path that will increase the model fit the greatest (i.e., outcome expectation to anticipated regret has a χ^2^ of 150.648), leading to the most significant improvement in model fit. The next path added was between cues to action and panic buying (i.e., χ^2^ of 34.961). Thereafter, no further modifications to the model were required.

**Figure 2:**
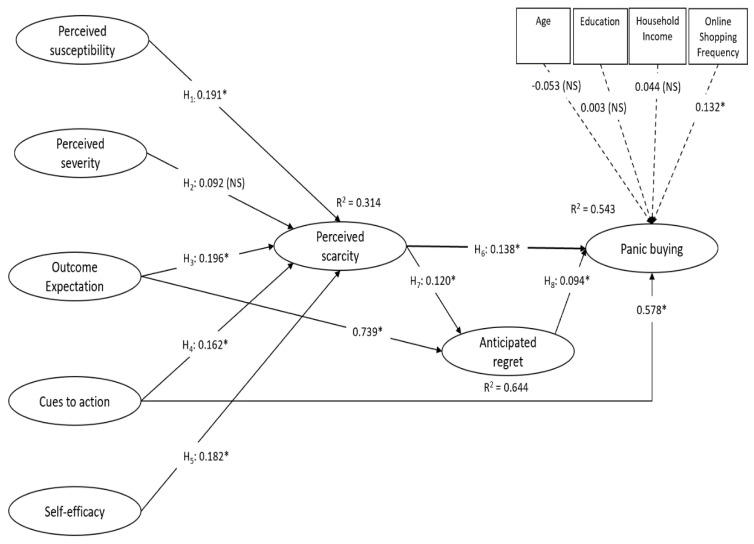
The proposed structural equation model after post hoc analysis. Note: * reflects that a path estimate is significant (*p* < 0.050); Model fit indices: χ^2^/df = 1.975, (*p* < 0.050, *df* = 351); CFI = 0.962; TLI = 0.956; RMSEA = 0.044; SRMR = 0.049.

In terms of explanatory strength, Figure 2 reflects that the structural model has a good model fit (χ^2^/df = 1.975, (*p* < 0.050, *df* = 351); CFI = 0.962; TLI = 0.956; RMSEA = 0.044; SRMR = 0.049). The squared multiple correlation (R^2^) values of the endogenous variables (i.e., perceived scarcity, anticipated regret, and panic buying) are more than 0.300, which shows the exogenous variable’s strong explanatory power.

The panic buying behavior is regressed on the control variables (i.e., ‘age’, ‘education’, ‘household income’, and ‘online shopping frequency’). The standardized regression estimates are −0.053, 0.003, 0.044, and 0.132, respectively. As seen, out of the four, only ‘online shopping frequency’ has a considerable influence on panic buying (*p* < 0.050)—the influence of ‘age’, ‘education’, and ‘household income’ are not significant. This research discovery is unexpected as younger people tend to perceive themselves to be less vulnerable in contracting COVID-19 and tend to take fewer preventive precautions to protect themselves from contracting COVID-19 (as reported in the United States), leading to less panic buying. However, this is found not to be the case in the present study. Furthermore, lower educated individuals are usually expected to be more susceptible to panic-inducing gossips and trends instead of being able to rationalize their buying decisions based on evidence-based news and research. Thus, it was expected that the less educated consumers would evaluate their purchasing needs based on an irrational fear sparked by their observation of other consumers’ panic buying behavior. However, this is not the case. Additionally, consumers with higher household income are also expected to engage more in panic buying behavior because of their larger purchasing power which allows them to spend more on each shopping trip compared to consumers with lower household income limited by their lower purchasing power. This expectation is aligned with Yoshizaki’s finding that panic buying is more prevalent among the more affluent people, albeit panic buying does not grow linearly with income but follows a concave function with a diminishing slope [132]. However, this is not the case in this survey. Only the result for ‘online shopping frequency’ is expected—the more often a consumer frequents the online shopping site, the more likely the consumer will be tempted to make an irrational and compulsive purchase. This is due to the increased likelihood to make a poor buying judgement because of the increased shopping accessibility and the number of cues to action (i.e., empty shelves and stockouts of products in limited supply). Therefore, consumers who have a higher online shopping frequency possess a higher tendency to panic buy. The comparatively weaker influence seen in the demographic variables concerning the theoretical factors shown in Figure 2 aligns with the results of Yuen et al. (2020). Their studies show that domain-specific attributes have a stronger causal relationship of panic buying behavior in relation to sociodemographic attributes (i.e., household income).

Four out of five of the health belief model components (i.e., perceived susceptibility, outcome expectation, cues to action, and self-efficacy) have a significant influence on consumers’ perception of scarcity. They have standardized effects of 0.191, 0.196, 0.162, and 0.182, respectively. Only perceived severity does not have a significant effect on perceived scarcity with a standardized effect of 0.092. This is likely because people are more concerned about contracting COVID-19 than the deadliness of the virus, given the low mortality rate in Singapore [133,134]. Even though perceived susceptibility and perceived severity are both perceived threats, but perceived susceptibility has a more significant effect on perceived scarcity than perceived severity.

Therefore, H_1_, H_3_, H_4_, H_5_ are accepted. H_2_ is not accepted. In combination with the control variables, these five variables explain 31.4% of the variance in perceived scarcity (R^2^ = 0.314). Generally, the outcomes corroborate the current study’s case that the health belief model variables lead to the perception of scarcity.

For example, perceived scarcity is influenced by consumers’ perceived susceptibility. If consumers perceive a lower risk of contracting COVID-19, the risk of freight movement restrictions is expected to decrease, increasing the replenishment rate of supply chain inventory, increasing the availability of products, and decreasing the risk of stockouts. Consequently, this decreases the perception of scarcity among consumers, decreasing the level of panic buying.

Similarly, outcome expectation is argued to influence perceived scarcity. Firstly, this is because of the perceived assurance that consumers will be protected from fluctuating prices. In a time of high price volatility, if consumers panic buy now as compared to buying later, they avoid the risk of paying a higher future price [51]. Thus, it leads to the decision to panic buy when the prices of goods are still relatively low. Because of this pursuit of perceived economic benefit, consumers’ perception of scarcity is exacerbated. Secondly, panic buying allows consumers to gain a perception of purpose and control in a time of high ambiguity as well as reduces stress and anxiety [3,21]. Thus, this pursuit of perceived hedonic benefit also exacerbates consumers’ perception of scarcity. In terms of functional utility, consumers perceive that panic buying allows them to be protected from the risk of stockouts and enables them to save time because of reduced commuting and shopping time, increasing their overall shopping convenience. Thus, it leads to more consumers pursuing this perceived functional benefit, leading to an increase in perceived scarcity. Lastly, there is an increased social utility from panic buying due to the social identity and acceptance that consumers gain from engaging in what everyone else from their online social media community is doing [90,91]. Hence, consumers’ pursuit for perceived social benefit leads to more consumers becoming more exposed to similar social media reports on scarce commodities, causing the increased perception of scarcity.

Additionally, cues to action influence consumers’ perception of scarcity through internal and external stimuli such as past trauma and broadcasting channels. Internally, consumers with a history of experiencing food insecurity will have their internal fears re-triggered, prompting them to perceive food scarcity due to past trauma. Externally, online and offline reports on limited supply (i.e., images of empty shelves) and increased demand (i.e., long queues of consumers buying large quantities of goods on a single trip) are cues that will trigger consumers to perceive a reduced availability of stock and a higher likelihood of a stockout situation, increasing their perception of scarcity.

Lastly, self-efficacy influences consumers’ perception of scarcity. This is because consumers with greater self-efficacy tend to have a more proactive self-preparation strategy towards risks. With a greater propensity to protect themselves from the risk of stockouts, these consumers are more likely to adopt a less risk-taking stance on the perceived level of product scarcity as a means to cope and protect themselves. Thus, they will take it upon themselves to view the risk of stockouts to be higher than consumers with lower self-efficacy. Consequently, their perceived extent of scarcity tends to be higher than consumers with a lower self-efficacy.

Figure 2 reflects that perceived scarcity will have a significant, positive impact on panic buying (*b* = 0.138, *p* < 0.05). Thus, H_6_ is accepted. This is aligned with perceived scarcity theory which proposes that consumers have a higher likelihood of engaging in panic buying if they perceive scarcity of goods and services to be high. With this in mind, a rational consumer will tend to panic buy because of the higher utility they perceive themselves gaining during a perceived scarcity situation.

Beyond the direct impact perceived scarcity has on panic buying, perceived scarcity also has an indirect impact on panic buying via the anticipated regret consumers foresee themselves experiencing. Perceived scarcity possesses a significant, positive impact on an individual’s anticipation of regret (*b* = 0.120, *p* < 0.05), leaving a significant, positive impact on panic buying (*b* = 0.094, *p* < 0.05). Thus, both H_7_ and H_8_ are accepted. The results validate the anticipated regret theory [67] which posits that panic buying is influenced by the consumer’s expectation that their failure to panic buy while they still can lead to a less desirable outcome than the outcome they would have enjoyed if they had engaged in panic buying [21]. Furthermore, anticipated regret can develop if the expected utility from anticipated regret increased or the negative outcome from anticipating regret decreased. Perceived scarcity can raise the expected utility from anticipated regret because it validates consumers’ increased sense of potential regret if they do not panic buy while they still can. Furthermore, specific attributes of perceived scarcity (e.g., increased perceived consumer rivalry and perceived price insecurity for available inventory) can create the perception among consumers that their risk of experiencing a negative outcome can be reduced by panic buying [51]. The above reasons explain the positive link between perceived scarcity and anticipated regret.

Finally, anticipated regret can influence an increase in panic buying behavior. Anticipated regret increases when there is greater uncertainty, leading to the tendency to engage in loss prevention [113]. Thus, this increases the likelihood for consumers to panic buy to avoid future regret (i.e., increased price and stockouts). In total, the theoretical model explains for 54.3% (R^2^ = 0.543). This is an improvement relative to the previous models that have R^2^ values between 0.134 and 0.220 [24,135], which is expected given that the theoretical model has an expanded scope with many of the variables in the existing models accounted for.

Beyond the original eight hypotheses proposed, the post hoc analysis highlighted that introducing two new paths (i.e., outcome expectation to anticipated regret and cues to action to panic buying) would improve the model fit significantly. First, outcome expectation has a significant impact on anticipated regret because consumers’ overall perception of the benefits gained from panic buying will translate to the amount of utility they foresee themselves foregoing if they do not panic buy while they still can. It has been noted that consumers anticipate regret if they do not make a purchase when prices are low during instances such as discounted/promotional sales [136,137]. Additionally, consumers also anticipate regret if they do not buy the product when they expect themselves to benefit from purchasing a product that is experiencing high demand, while they still can [138,139]. In other words, the amount of perceived benefits consumers assign to panic buying is the extent of potential regret they foresee themselves experiencing due to the foregone benefits they could have gained.

Second, cues to action are directly associated with panic buying. For external cues to action, the exchange of information on social networking sites allows for data points on the panic buying situation, that serve as social proof of society panic buying, to be shared globally [19]. It provides consumers with proof of other consumer’s purchasing insecurities, proof of friends’ engagement with panic buying as a means to cope, proof of goods in limited supply, proof of policymakers’ pandemic management plans, and social proof of experts advice on the situation [140]. This consequently affects consumer values and consumer socialization, which influences consumers’ buying decisions [141,142]. For internal cues to action, past experience related to product scarcity will trigger consumers to engage in panic buying [75]. Overall, information sources have been shown to influence consumer behavior significantly over the recent disease outbreak [143]. Thus, drawing a direct path between cues to action and panic buying is an accurate illustration of their interrelationship with each other in the theoretical model.

### 4.3. Direct, Indirect, and Total Effect Analysis

Perceived scarcity partially mediates the influence of the five health belief model components on panic buying. Additionally, anticipated regret partially mediates the influence of perceived scarcity on panic buying. As seen in Figure 2, every exogenous variable has a significant total effect on panic buying except for perceived severity.

Table 6 shows the exogenous variables’ direct, indirect, and total effects on the endogenous variables. In terms of direct effects, the most important determinants of perceived scarcity start with outcome expectation (a_31_ = 0.196), followed by perceived susceptibility (a_11_ = 0.191), self-efficacy (a_51_ = 0.182), cues to action (a_41_ = 0.162), and perceived severity (a_21_ = not significant). The two direct predictors of anticipated regret are perceived scarcity (a_62_ = 0.120) and outcome expectation (a_32_ = 0.739). Finally, the direct predictors of panic = buying are cues to action (a_43_ = 0.578), perceived scarcity (a_63_ = 0.138), and anticipated regret (a_73_ = 0.094).

In terms of indirect effects, the most important predictors of anticipated regret are outcome expectation (b_32_ = 0.024), followed by perceived susceptibility (b_12_ = 0.023), self-efficacy (b_52_ = 0.022), cues to action (b_42_ = 0.019), and perceived severity (b_22_ = not significant). As seen in Figure 2, the health belief model variable’s influence on panic buying is partly mediated by perceived scarcity and partly by anticipated regret. The strongest effect on panic buying is from outcome expectation (b_33_ = 0.099), followed by perceived susceptibility (b_13_ = 0.029), self-efficacy (b_53_ = 0.027), cues to action (b_43_ = 0.024), and perceived severity (b_23_ = not significant).

In terms of total effects, cues to action (c_43_ = 0.602) possesses the greatest total effect on panic buying. The second would be perceived scarcity (c_63_ = 0.138), followed by outcome expectation (c_33_ = 0.099), anticipated regret (c_73_ = 0.094), perceived susceptibility (c_13_ = 0.029), self-efficacy (c_53_ = 0.027), and lastly perceived severity (c_23_ = not significant).

## 5. Conclusions

### 5.1. Summary

This study’s objective is to identify the determinants of panic buying and understand their interrelationships. Using the health belief model, perceived scarcity theory, and anticipated regret theory, this study offers a theoretical model that describes the determinants of panic buying. The key position is that panic buying is directly affected by both perceived scarcity and anticipated regret. Furthermore, perceived scarcity has a positive effect on panic buying because consumers’ heightened perception of the limited availability of stocks causes them to foresee a risk of regretting a lost opportunity to purchase products in limited supply while they still can. Thus, it prompts them to act upon this increased urgency to panic buy. Additionally, perceived scarcity can be supported by the components of the health belief model which proposes five health belief model factors that lead to the perception of scarcity.

A survey was conducted in Singapore through a professional survey firm using the firm’s online channels. The survey received 508 valid data points. The outcomes indicate that perceived scarcity mediates the relationship between the health belief model variables (i.e., perceived susceptibility, perceived severity, outcome expectation, cues to action, and self-efficacy) and panic buying. Additionally, the perceived scarcity impact on panic buying is partially mediated by anticipated regret. The total effect analysis reflects that perceived scarcity has the largest impact on panic buying. The remaining factors, in decreasing order of impact, are anticipated regret, outcome expectation, cues to action, perceived susceptibility, self-efficacy, and perceived severity.

### 5.2. Theoretical Contributions

This paper has contributed significantly to the academic research field. First, the paper addresses a missing link in the study on panic buying by applying and adapting three theories, namely, the health belief model theory, perceived scarcity theory, and anticipated regret theory, to identify and analyze the factors affecting panic buying behavior. The current theoretical panic buying research is very limited. Within which, most research employs the stimulus-organism-response model [144], protective action decision model [145], competitive arousal model [144], or its extension by infusing constructs such as fear or trust [146] and herding behavior or observational learning [147]. This research paper expands on the work of [67] and [21] and offers an alternative analysis of determinants for panic buying. For example, [67] integrated perceived scarcity and anticipated regret anchored on competitive hedonic motivations and [21] synthesized a model based on the perceived threat, fear, coping methods, and social psychological factors. Despite some overlapping similarities, this paper has analyzed and utilized other theories such as the health belief model. These theories (i.e., health belief model, perceived scarcity, anticipated regret) are founded on diverse paradigms such as psychological, decision making, and abundance-scarcity research to offer a comprehensive study on the determinants of panic buying.

Furthermore, this paper contributes to existing research by identifying, proposing, and operationalizing the various determinants of panic buying, namely, perceived susceptibility, perceived severity, outcome expectation, cues to action, and self-efficacy. These factors represent a considerable percentage of the panic buying variance (54.3%). This implies that a mixture of theories has a stronger explanatory power than a single theory, as often seen in research explaining panic buying. More importantly, the results suggest that the theories complement each other in explaining the determinants of panic buying. Even so, it is important to note that the sufficiency of each theory in explaining panic buying differs. According to the analysis of the total effects, the health belief model theory possesses the highest explanatory ability. The second would be perceived scarcity theory and the last would be anticipated regret theory. Additionally, this paper offers a clearer nomological appreciation of the relationships among the variables affecting panic buying. The results are in alignment with the paper’s key arguments that consumers’ perceived susceptibility, perceived severity, outcome expectation, cues to action, and self-efficacy are factors influencing their decision to panic buy.

Complementarily, perceived scarcity, anticipated regret, and cues to action correlates to the decision to panic buy. This conclusion is aligned with the key principles of motivation research which states that the decision process of consumers starts with the evaluation of their perception of scarcity, which leads to the anticipation of regret. Consequently, the perception of scarcity directly or indirectly leads to the decision to panic buy. Additionally, Laato et al. (2020) has also noted how cues to action, through various information sources, can directly influence consumers’ buying decision.

In conjunction, this paper contributes to the quantitative assessment or operationalization of the constructs. The measurement items were designed by synthesizing the health belief model, perceived scarcity theory, and anticipated regret theory constructs adapted from research papers on the pandemic to ensure their fit in the context of panic buying. To some extent, this paper has given a clearer explanation of the impact of perceived scarcity on panic buying. Past research has offered an ambiguous stance on the influence of perceived scarcity on panic buying. To illustrate, [22] found that perceived scarcity has a strong link with panic buying. At the same time, [144] proposed that perceived scarcity is linked to panic buying indirectly through a psychological construct known as perceived arousal. Finally, some others noted that perceived scarcity motivates panic buying and is directly and indirectly linked to panic buying via anticipated regret [21,148]. The results of this paper align with some of the past research which detected significant direct and indirect relationships between perceived scarcity and panic buying. However, in contrast to previous research, the current paper suggests that perceived scarcity is influenced by the dimensions of the health belief model theory. The notion that perceived scarcity is influenced by evaluating the health belief model components is aligned with other health protection research such as water conservation [81] and breast cancer early diagnosis [149]. This paper suggests that there must be some basis for a consumer to anticipate regret. Thus, anticipated regret will be developed if consumers constantly perceive a product as scarce. Perceived scarcity is in turn affected by consumers’ assessment of the health belief model components such as perceived susceptibility, perceived severity, outcome expectation, cues to action, and self-efficacy.

### 5.3. Policy Implications

To address policy concerns, this paper proposes some recommended solutions for policymakers to manage perceived scarcity to resolve the panic buying situation. Relative to rational buying, panic buying confers many disadvantages and detriments to various stakeholders including food producers, supply chain transport operators, retail store managers, society, and the environment. Relevant stakeholders need to understand the importance of managing the perceived scarcity of consumers to better manage panic buying. Additionally, to a large extent, perceived scarcity generates anticipated regret in consumers, leading to the decision to panic buy.

The study of the total effects on panic buying shows that resources should first be assigned to minimizing consumer’s exposure to cues to action. Thus, to reduce the number of triggers prompting consumers to panic buy, the media can consider showing more images of fully-stocked shelves instead of empty ones; community and religious leaders could consider minimizing the number of times they mention the panic buying phenomenon to reduce the number of times the perception of scarcity is reinforced in consumers’ minds; family and friends could consider giving each other mental health protection prompts instead of prompts to panic buy products.

The next biggest priority would be to reduce the consumer’s perception of scarcity. To do so, governments can consider enforcing a limit on the number of critical stocks each consumer can purchase at a go, directly taking the pressure off products in scarce supply. Additionally, retail shops can increase the frequency of restocking shelves to prevent incidences of empty shelves as well as give priority to the vulnerable groups in society so that there is a decrease in their perceived susceptibility to contracting COVID-19. Panic buying is frequently executed by consumers that fear stockouts due to their perception of scarcity, thus, reducing consumers’ perceived scarcity will reduce their panic buying behavior as well. The evaluation of perceived scarcity is a subjective judgement, so policymakers can invest in marketing campaigns that raise awareness on the actual level of readiness of available national stockpiles to convince consumers that the level of scarcity is not as high as they initially perceived it to be.

Thereafter, policymakers and retail owners can consider working on other variables which, in order of descending importance, are outcome expectation, anticipated regret, perceived susceptibility, self-efficacy, and perceived severity. For outcome expectation, retail owners can stock shelves with items that have a more immediate expiry date such that the benefit of panic buying will be reduced due to the earlier expiration of food items. Additionally, retail owners can stock shelves more frequently but in smaller amounts each time to ensure that the likelihood of a stockout and appearance of empty shelves will be reduced. To reduce the perceived benefit that panic buying lowers the risk of contracting COVID-19 due to less frequent store visits, retail stores can limit the number of people shopping in the store at any given moment as well as disinfect the stores routinely to ensure that the likelihood of contracting COVID-19 from a shopping trip is reduced. Socially, community leaders, friends, and families could discourage and condemn the socially inconsiderate act of panic buying as it deprives others in the community of products in limited supply.

Next, anticipated regret can be reduced through refocusing consumer’s attention and anticipation of regret away from panic buying products towards more productive and effective preparation methods. This can be done through policymakers educating the public on accurate and effective ways to prepare for COVID-19 such as maintaining physical (i.e., doing regular exercise, getting enough sleep), mental (i.e., managing stress and anxiety through meditation), and emotional (i.e., checking in on friends and family) well-being, instead of panic buying.

For perceived susceptibility, policymakers can institute rules on social distancing and mandatory mask-wearing to reduce the population’s general risk of contracting COVID-19. Individually, consumers can also try to lead a healthy lifestyle that maintains their physical, mental, and emotional well-being. For self-efficacy, although there is a positive impact on perceived scarcity, it is a healthy trait to have during this pandemic to ensure resilience and self-protection, thus, there is no recommendation to reduce perceived scarcity from this dimension. Lastly, for perceived severity, employers can consider assuring their employees that their employment will not be affected if they contract COVID-19; family and friends can regularly check in on each other to give emotional assurance and support to each other, and individuals can remind themselves that the healthcare system is robust and the mortality rate is lower than 1% if they ever contract it. This reduces the perceived severity of contracting COVID-19, causing the perceived scarcity by individuals to reduce as well, eliciting a reduction in panic buying.

### 5.4. Limitations and Recommendations

This research paper has six main limitations to consider. First, the study is carried out in Singapore—a city-state with a population density of 8291.9 inhabitants per square km (i.e., the third most densely populated country worldwide in 2019) [150] that is mostly reliant on the importation of essential and non-essential supplies. Additionally, the degree of uncertainty avoidance is one of the highest in Singapore [26,27], thus, these results may differ for other countries with a different degree of uncertainty avoidance. Therefore, it is advised to interpret the results bearing in mind that it does not apply to other contexts, for example, in rural or suburban locations. Perception of scarcity may have a stronger impact in more densely populated regions that are heavily reliant on trade such as Singapore, especially when trade volatility increases. This enables a greater perception of scarcity and triggers stronger anticipation of regret if consumers do not panic buy while they still can. Therefore, future studies could further examine the research model’s generalizability by cross-referencing it to different situations.

The second limitation is that the paper simply proposed three theoretical perspectives to analyze and account for the determinants of panic buying. An extension of this study can look into offering new theories on or studying the distinctions (i.e., moderating effects) in panic buying.

The third limitation is about the method used to collect the data. As the survey was done online, the survey participants are mostly literate and computer-savvy consumers. Thus, they would be well-informed on the news and more susceptible to online cues to action as compared to consumers that are not computer-savvy. Thus, further study can be done through offline methods to ensure that the data collated is representative of the entire population—computer-savvy and not computer-savvy.

The fourth limitation is the participant profiles collected. The survey conducted did not capture the participants’ nature of occupations. There is a possibility that an essential worker needing to work long hours may panic buy very differently from those with the flexibility to work from home. Workers with the flexibility of working from home may have the freedom of time to panic buy more frequently, but essential workers working shift work and long hours may only be able to panic buy in limited window periods after they end work. Therefore, this paper acknowledges the potential effect of different occupation requirements and flexibility on the panic buying behavior of consumers and recognizes that further study can be done to uncover the relationship between consumers’ nature of work and panic buying behavior.

The fifth limitation is the inability to test the validity of the relationship of perceived scarcity’s effect on perceived severity. Perceived severity’s effect on perceived scarcity is not significant, and this could be because of the presence of a mediator or that the relationship is reversed. However, structural equation modeling is unable to validate these probable relationships. Thus, further experiments can be done to understand this possible relationship better.

Lastly, the study on panic buying is still in its nascent stage as the COVID-19 pandemic is a recent event. Thus, the existing research on this issue is still relatively limited. Furthermore, the panic buying issue is still an ongoing incident overseas, thus, it would be important to continue to analyze the future developments surrounding this relatively new global phenomenon.

## Figures and Tables

**Table 1:** A review of belief theories and factors affecting panic buying behavior.

Theory’s Characteristics	Health Belief Model	Perceived Scarcity Theory	Anticipated Regret Theory
Basic assumption	The health belief model’s representative constructs can explain health protection behavior [42,43,44].	A negative perception of the availability of goods and services leading to an evaluation of limited stock could lead to panic buying [39,40].	Considerations of the expected negative psychological and emotional result, regret, when making a decision could lead to panic buying in an attempt to avoid the undesirable stockout outcome in the future [45,46,47].
Underlying constructs	Perceived susceptibility, perceived severity, outcome expectation, cues to action, and self-efficacy [43,48,49]	Perceived scarcity	Anticipated regret
Specific contributions to model	The theory can justify how its representative constructs lead to perceived scarcity by leaving an impression of limited merchandise supply and availability [50].	The theory can describe how perceived scarcity contributes to anticipated regret [51], which subsequently leads to panic buying [52,53].	The theory can expound how consumers’ anticipated regret results in panic buying behavior [46,54].

**Table 2:** Measurement items and constructs.

Construct	ID	Measurement Items	Modified Source
Perceived Susceptibility		*Range between 1 (strongly disagree) to 7 (strongly agree)*	
	SUS1	My chance of contracting COVID-19 is greater than others	[118]
	SUS2	Due to my physical health, I would more probably contract COVID-19	[123]
	SUS3	I feel that my probability of contracting COVID-19 in the future is high	
Perceived Severity		*Range between 1 (strongly disagree) to 7 (strongly agree)*	
	SEV1	The thought of contracting COVID-19 scares me	[119]
	SEV2	If I had COVID-19, my career would be endangered	
	SEV3	If I had COVID-19, my relationships with my family and friends will be affected	
Outcome Expectation		*Range between 1 (strongly disagree) to 7 (strongly agree)*	
	OUT1	Stockpiling products will be beneficial	[119];
	OUT2	Stockpiling products protects me from a stock-out situation	[124]
	OUT3	Stockpiling products reduces my risk of contracting COVID-19 by minimizing visits to the stores or crowds	
Self-efficacy		*Range between 1 (strongly disagree) to 7 (strongly agree)*	[125];
	SEL1	I am confident that I can protect myself from COVID-19	[118];
	SEL2	I possess knowledge about protecting myself from COVID-19	[78];
	SEL3	Professional information about protecting myself from COVID-19 is searchable and available	[123]
Cues to action		*Range between 1 (strongly disagree) to 7 (strongly agree)*	
	CUE1	My family prompted me to stockpile products at home	[119]
	CUE2	My previous experience prompted me to stockpile products at home	
	CUE3	My friends prompted me to stockpile products at home	
	CUE4	The media prompted me to stockpile products at home	
Perceived scarcity		*Range between 1 (strongly disagree) to 7 (strongly agree)*	
	SCA1	The products that I feel the want to buy will be very limited during COVID-19	[121]
	SCA2	The brand availability for a product will be very limited during COVID-19	
	SCA3	The sizes of a product will be very limited during COVID-19	
Anticipated regret		*Range between 1 (strongly disagree) to 7 (strongly agree)*	
	REG1	If I do not stockpile products, I would regret later	[126];
	REG2	If I do not stockpile products, I would feel sorry about my choice later	[67]
	REG3	If I do not stockpile products, I would feel that I had not done enough to prepare for COVID-19	
Panic buying		*Range between 1 (strongly disagree) to 7 (strongly agree)*	
	PB1	I had the urge to grab products immediately	[121]
	PB2	I snapped things up during the shopping trip in this shop	
	PB3	When I took a product, I did not want to place it down even though I was not certain if I would purchase it or not	

**Table 3:** Respondent’s profile.

Characteristics	Frequency	Proportion (%)
**Gender**		
Female	247	49
Male	261	51
**Age (years)**		
16–34	247	49
35–49	193	38
50 and above	68	13
* Education		
Primary	1	0
Secondary & Pre-university	75	15
Tertiary	432	85
**Housing**		
Private housing	113	22
Public housing	395	78
* Household income (Singapore Dollar (SGD)/month)		
0–7999	282	55
8000–19,999	201	40
20,000 and above	25	5
* Online Shopping Frequency		
Almost Never	2	0
Few times a year	85	17
Few times a month	258	51
Few times a week	139	27
Daily	24	5

* Variable used as a control factor in the theoretical model.

**Table 4:** Confirmatory factor analysis results.

Construct	Item	λ	AVE	CR
Perceived Susceptibility	SUS1	0.850	0.734	0.892
	SUS2	0.843		
	SUS3	0.876		
Perceived Severity	SEV1	0.679	0.534	0.773
	SEV2	0.831		
	SEV3	0.670		
Outcome Expectation	OUT1	0.753	0.716	0.883
	OUT2	0.881		
	OUT3	0.897		
Self-efficacy	SEL1	0.747	0.644	0.843
	SEL2	0.913		
	SEL3	0.735		
Cues to action	CTA1	0.825	0.749	0.922
	CTA2	0.944		
	CTA3	0.903		
	CTA4	0.780		
Perceived scarcity	SCA1	0.809	0.731	0.891
	SCA2	0.910		
	SCA3	0.843		
Anticipated regret	REG1	0.892	0.783	0.915
	REG2	0.891		
	REG3	0.871		
Panic buying	PB1	0.842	0.680	0.864
	PB2	0.839		
	PB3	0.791		

Note: Model fit indices χ^2^/df = 2.064, (*p* < 0.050, *df* = 247); CFI = 0.970; TLI = 0.964; RMSEA = 0.050; SRMR = 0.030.

**Table 5:** AVE, correlations, and squared correlations of the constructs.

	SUS	SEV	OUT	SEL	CUE	SCA	REG	PB
SUS	0.734 ^a^	0.262 ^c^	0.183	0.008	0.245	0.148	0.215	0.236
SEV	0.512 ^b^	0.534	0.243	0.011	0.309	0.156	0.218	0.201
OUT	0.428	0.493	0.716	0.007	0.582	0.217	0.596	0.352
SEL	−0.090	0.104	0.084	0.644	0.001	0.038	0.001	0.011
CUE	0.495	0.556	0.763	0.024	0.749	0.216	0.526	0.510
SCA	0.385	0.395	0.466	0.194	0.465	0.731	0.214	0.204
REG	0.464	0.467	0.772	0.033	0.725	0.463	0.783	0.339
PB	0.486	0.448	0.593	0.104	0.714	0.452	0.582	0.680

^a^ AVE values are along the main diagonal. ^b^ Correlations between constructs are under the main diagonal. ^c^ Squared correlations between constructs are above the main diagonal.

**Table 6:** Direct, indirect, and total effects.

Exogenous (i)	Endogenous (j)		
	Perceived Scarcity (1)	Anticipated Regret (2)	Panic Buying (3)
**Direct effects (a_ij_) of …**			
Perceived susceptibility (1)	0.191	-	-
Perceived severity (2)	-	-	-
Outcome expectation (3)	0.196	0.739	-
Cues to action (4)	0.162	-	0.578
Self-efficacy (5)	0.182	-	-
Perceived scarcity (6)	-	0.120	0.138
Anticipated regret (7)	-	-	0.094
**Indirect effects (b_ij_) of …**			
Perceived susceptibility (1)	-	0.023	0.029
Perceived severity (2)	-	-	-
Outcome expectation (3)	-	0.024	0.099
Cues to action (4)	-	0.019	0.024
Self-efficacy (5)	-	0.022	0.027
Perceived scarcity (6)	-	-	-
Anticipated regret (7)	-	-	-
**Total effects (c_ij_) of …**			
Perceived susceptibility (1)	0.191	0.023	0.029
Perceived severity (2)	-	-	-
Outcome expectation (3)	0.196	0.763	0.099
Cues to action (4)	0.162	0.019	0.602
Self-efficacy (5)	0.182	0.022	0.027
Perceived scarcity (6)	-	0.120	0.138
Anticipated regret (7)	-	-	0.094

## Data Availability

Restrictions apply to the availability of these data. Data was obtained from Qualtrics and are available from the authors with the permission of Qualtrics.

## Appendix A

**Table A1:** The CHERRIES Checklist.

Item Category	Checklist Item	Explanation
Design	Describe survey design	The target population is the entire Singapore consumer population, and the sample was designed to be representative of that based on certain demographic characteristics such as age, gender, income and type of housing. The survey is administered in English.
IRB (Institutional Review Board) approval and informed consent process	IRB approval	Ethical review was exempted for this study, due to anonymous educational tests, surveys, interviews, and public observations. The nature of the study falls under Exempt Category 2 (http://research.ntu.edu.sg/rieo/IRB/Guidelines/Pages/Exempt-cat-2.aspx accessed on 10 March 2021)
	Informed consent	Consent was obtained from the participants prior to survey administration. Respondents must be at least 16 years old to participate.
	Data protection	The data were password-encrypted and stored in a hard drive which only the investigators have access to. Information that identifies the respondents was not collected.
Development and pre-testing	Development and testing	A blended partnering panel approach was taken to ensure that the sampling frame is representative of the population. In the beginning, the survey underwent a soft launch, whereby a small sample of responses was collated, to make minor improvements to the survey.
Recruitment process and description of the sample having access to the questionnaire	Open survey versus closed survey	Open survey
	Contact mode	The engaged survey company sent out questionnaires by email to the participants whose responses are provided via an online survey.
	Advertising the survey	No advertisement
Survey administration	Web/E-mail	E-mail
	Context	Survey questions are available on the survey company’s webpage. The survey was administered in English
	Mandatory/voluntary	Voluntary survey
	Incentives	There were monetary incentives offered to each participant (approximately 3–5 USD per person).
	Time/Date	Data was collected over 18 days from 26 June to 13 July 2020. The average completion time is about 8 min.
	Randomization of items or questionnaires	Identical items but in a reversed manner are included in the survey to validate the participants’ responses
	Adaptive questioning	No adaptive questioning
	Number of Items	The survey contains 15 questions each with multiple items and a few socio-demographic questions.
	Completeness check	Respondents must complete all questions before they can submit the survey
	Review step	Respondents were able to review their answers
Response rates	Unique site visitor	Information not available
	View rate (ratio of unique survey visitors to unique site visitors)	Information not available
	Participation rate (ratio of unique visitors who agreed to participate to unique first survey page visitors)	Information not available
	Completion rate (ratio of users who finished survey to users who agreed to participate)	508/1700 = 29.88%
Preventing multiple entries from the same individual	IP check	Each participant’s IP is logged to prevent multiple attempts
Analysis	Handling of incomplete questionnaires	Not applicable. Respondents must complete all questions before they can submit the survey
	Questionnaires submitted with an atypical timestamp	The time taken and time of completion are recorded
	Statistical correction	There is no weighting of items or propensity scores
